# Isolated Left Ventricular Noncompaction Presenting With Heart Failure With Reduced Ejection Fraction and Intrahospital Cardiac Arrest: A Case Report and Literature Review

**DOI:** 10.7759/cureus.30559

**Published:** 2022-10-21

**Authors:** Filipa Madalena F Gonçalves, Marta Batista, Ana Luísa Campos, Magda Costa, Jorge Cotter

**Affiliations:** 1 Internal Medicine, Hospital da Senhora da Oliveira, Guimarães, PRT

**Keywords:** cardiomyopathy, left ventricular noncompaction, ventricular trabeculations, cardiac arrest, heart failure, non-compaction, left ventricle

## Abstract

Left ventricular noncompaction (LVNC) is characterized by a bilayered appearance of the myocardium with excessive trabeculations and deep intertrabecular recesses. Manifestations of this condition are widely variable, ranging from incidental findings in asymptomatic individuals to symptomatic heart failure, conduction abnormalities, tachyarrhythmia, and sudden cardiac death. Heart failure, ventricular arrhythmias, and systemic embolisms are the most frequent cardiovascular complications.

We describe a case of a 53-year-old woman who presented to the emergency department with acute presentation of previously unknown heart failure with reduced ejection fraction and was diagnosed with LVNC. During hospitalization, the patient presented a defibrillated cardiac arrest rhythm, which was resuscitated after six minutes, and then treated with the placement of an implantable cardioverter defibrillator. After two years of follow-up with optimized medical therapy, the patient currently is asymptomatic and with a preserved ejection fraction.

## Introduction

The pathogenesis of left ventricular noncompaction (LVNC) has been regarded as an arrest in myocardium compaction during embryogenesis due to genetic causes [1]. It is characterized by a bilayered appearance of the myocardium, with excessive trabeculations and deep intertrabecular recesses [2].

The main cardiac symptoms are related to heart failure (HF), which occurs in half of the patients; atrial fibrillation and ventricular tachyarrhythmias are also frequent [3]. LVNC is managed like cardiomyopathy from other etiologies [4]. The prognosis of patients can vary according to structural and hemodynamic complications [5].

## Case presentation

**Figure 1:**
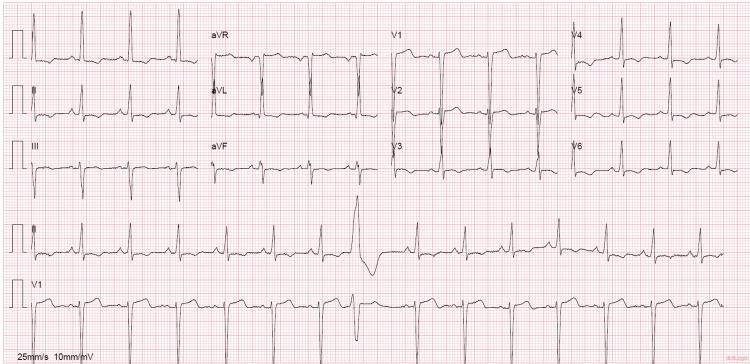
ECG at admission ECG revealed sinus rhythm with premature ventricular contraction, with ventricular repolarization alterations suggestive of overload and/or ischemia.

**Figure 2:**
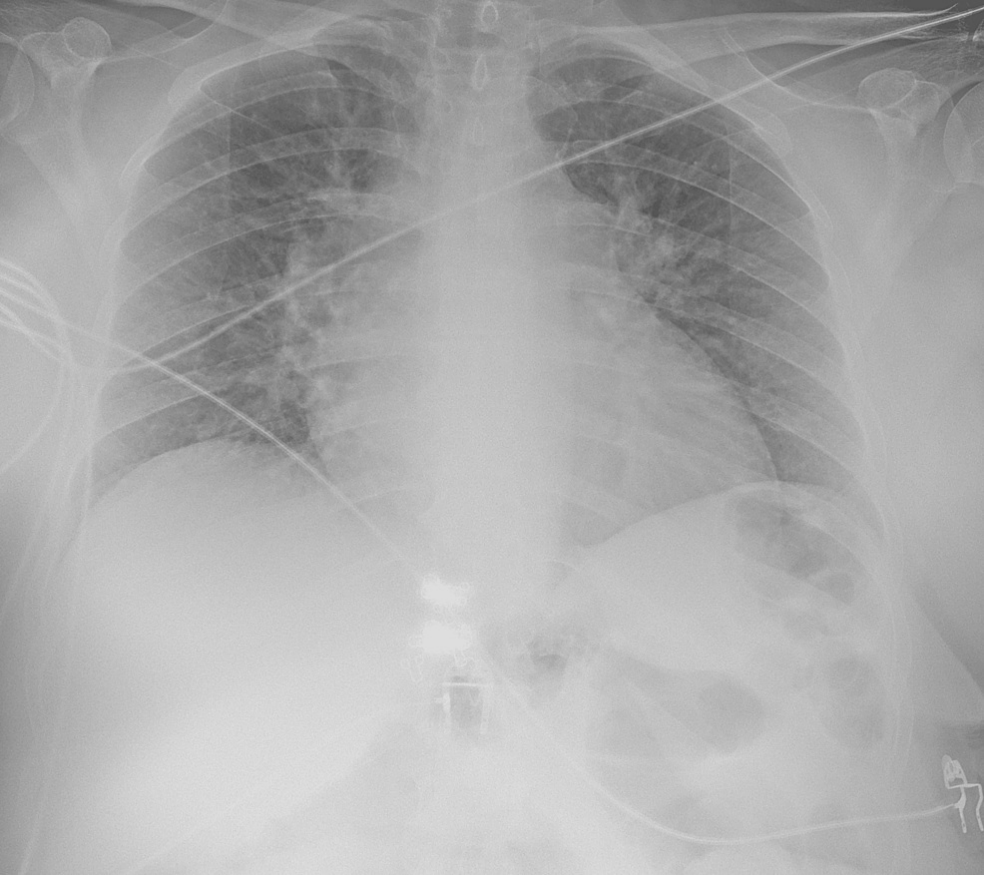
Chest radiograph (single view) Chest radiograph in a posteroanterior incidence showed cardiomegaly. There are no consolidations and both costophrenic angles are seen.

**Figure 3:**
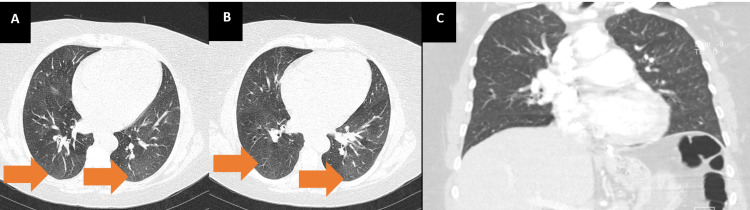
CT scan Images (A) and (B) show the axial planes of the CT scan. Arrows showing ground glass areas in the dependent part of the lungs, corresponding to lung congestion. Image (C) shows the coronal plane with no consolidation.

A 53-year-old woman with a past medical history of hypertension, smoking, asthma, and obesity, with previous gastric bypass surgery eight years before, presented to the emergency department (ED) with shortness of breath on minimal exertion for two months, worsened in the six days prior to admission and progressing to dyspnea at rest, and orthopnea, associated with scanty dry cough. The patient denied fever, myalgias, chest pain, paroxysmal nocturnal dyspnea, and lower limb swelling. On admission to the ED, the patient had a blood pressure of 130/60 mmHg, pulse rate of 99 beats/minute, regular rate and rhythm, a body temperature of 37.8ºC, pulse oximetry of 96% at FiO_2_ 21%, and respiratory rate of 22 breaths per minute. The physical examination showed positive hepatojugular reflux, without jugular vein distention at 45º, no murmurs were identified on cardiac auscultation, and diffuse bilateral inspiratory wet pulmonary crackles and moderate wheezes were heard on pulmonary auscultation. Laboratory evaluation showed hypochromic microcytic anemia, normal kidney, liver, and thyroid function, elevated C-reactive protein (102 mg/L), and no elevation in myocardial injury markers, although an elevated B-natriuretic peptide (BNP) level of 4270 pg/mL was noted. An electrocardiogram (ECG) (Figure 1) and a chest X-ray (Figure 2) were performed. A pulmonary angio-CT scan excluded pneumonia and pulmonary thromboembolism or other major vascular complications (Figure 3).

Due to decompensated non-stratified HF diagnosis and a community-acquired tracheobronchitis, empiric antibiotic therapy was initiated with amoxicillin/clavulanic acid and azithromycin and the patient was admitted to the internal medicine unit.

An echocardiogram performed during admission revealed severe dilatation of the left ventricle, with left ventricular ejection function (LVEF) estimated at 31%, and also described as left eccentric ventricular hypertrophy; inferior vena cava was not dilated. During hospitalization, the patient remained symptomatic despite high-dose diuretics, associated with an increase in BNP of 10712 pg/mL.

**Figure 4:**
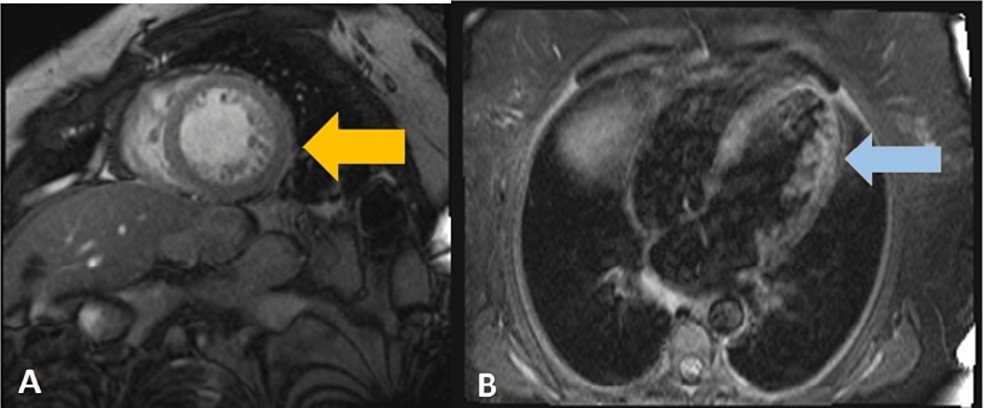
Cardiac magnetic resonance Cardiac magnetic resonance imaging showed left ventricular hypertrabeculation and deep myocardial intratrabecular recesses (arrows). The ratio of the compacted to noncompacted myocardium was 2.75. (A) Axial view with short axis plan. (B) Axial view with long axis plan.

Cardiac magnetic resonance imaging (MRI) (Figure 4) was performed on the 12th day of hospitalization. It showed prominent trabeculation of the anterolateral and inferolateral walls of the left ventricle, with a maximum end-diastolic noncompacted to compacted myocardial thickness ratio of 2.75; it also excluded changes in the right ventricle, ischemic, or fibrotic areas. The diagnosis of dilated cardiomyopathy with phenotypic characteristics of noncompaction of the left ventricle was made. Perfusion MRI study demonstrated the absence of significant ischemic disease.

The following day, a cardiorespiratory arrest was witnessed and resuscitated promptly. Ventricular fibrillation was identified and a shock was administered, with spontaneous circulation recovery after, with a total arrest time of six minutes. The patient was admitted to a cardiac care unit and an implantable cardioverter defibrillator (ICD) was placed. When clinically stable, therapy with an angiotensin receptor-neprilysin inhibitor (ARNI) was initiated. After 19 days of admission, the patient was discharged and referred to a cardiology consultation. Smoking cessation was advised.

The genetic study was negative and included a search for mutations in the following genes: LIM domain-binding protein 3, α-dystrobrevin, tafazzin, MYH7, ACTC1, TNNT2, TPM1, TNNI3, and MYBPC3. The patient started anticoagulation due to a deep vein thrombosis.

During the first 16 months of follow-up under optimized tolerance-based ARNI, bisoprolol, and spironolactone therapy, an improvement in left ventricular function was achieved with transthoracic echocardiography revealing an ejection fraction of 55%.

## Discussion

LVNC is characterized by the excessive formation of trabeculae and intertrabecular recesses in the uncompressed inner myocardial wall associated with the mesocardium, mostly affecting the apical region of the left ventricle, but in rare cases, it can affect the right ventricle [3]. It was first described in 1926, and has a reported prevalence of 0.014-0.17%, with a higher prevalence in male patients (52-86% of cases) [3,5]. In patients with HF, the prevalence of ventricular noncompaction was reported to be 3% [3].

The main cause of LVNC is the mutation of genes encoding sarcomeric proteins, but also mutations in genes encoding cytoskeletal, Z-line, and mitochondrial proteins may be identified [3]. The most commonly mutated genes are MYH7, MYBPC3, and TTN [5]. LVNC is an uncommon primary genetic cardiomyopathy, so first-degree relatives should undergo a screening process [3,4]. LVNC may be associated with congenital heart diseases, coronary artery anomalies and conotruncal anomalies, septal defects, anomalous venous pulmonary veins, hypoplastic left heart syndrome, and Ebstein anomaly [6].

Nine subtypes are described: isolated or benign, the arrhythmogenic form, the dilated form, the hypertrophic form, the “mixed” form, the restrictive form, the biventricular form, the right ventricular hypertrabeculation with normal left ventricular (LV) form, and the congenital heart disease form [7]. In some cases, there may be an overlap with another myocardial disease (hypertrophic, dilated, or restrictive cardiomyopathy) [8].

There is a mismatch between myocardial mass and the number of capillaries, leading to hypoperfusion of the endocardium, despite normal epicardial coronary arteries, with hypokinetic areas observed both in compacted and noncompacted segments [9]. Also, trabeculations diminish ventricular compliance, promoting diastolic dysfunction; systolic dysfunction is attributed to hypoperfusion secondary to subendocardial microvascular abnormalities and by LV dyssynchrony between the compacted and noncompacted myocardial layers [10].

Initial ECG’s most common findings are intraventricular conduction delay, including left branch block, auriculoventricular block, and repolarization abnormalities such as QTc prolongation; also, LV hypertrophy is often present, although it can be normal in 13% of cases [5,9,10]. ECG abnormalities are strongly associated with LNVC, with repolarization disturbances predisposing patients to malignant ventricular arrhythmias that can lead to sudden cardiac death [10]. Transthoracic echocardiography is the first step in the diagnosis, although it is difficult to identify trabeculations, and may underestimate the degree of LVNC. Cardiac MRI (CMRI) and left ventriculography are other imaging examinations that may be useful [3]. The best criterion for diagnosis of LVNC in CMRI was a ratio of the noncompacted to compacted myocardium of >2.3 in diastole [3]. The most commonly used criteria for echocardiographic diagnosis were proposed by Jenni et al., which consider a maximum ratio between uncompressed and compacted myocardium greater than 2 at the end of the systole parasternal short axis, and the middle ad apical ventricular segments should be involved, with evidence of ventricular cavity blood flow in deep intertrabecular recesses by color Doppler [3]. For risk stratification, nuclear MRI detects the presence of focal myocardial fibrosis, which is related to ventricular arrhythmias, lower ejection fraction, and worse prognosis [3].

Manifestations of this condition are widely variable, ranging from incidentally identified cardiac findings in asymptomatic individuals with or without cardiovascular risk factors to symptomatic HF, conduction abnormalities, tachyarrhythmia, and sudden cardiac death [10]. Malignant arrhythmia or sudden cardiac arrest is a rare presentation, representing 2-5% of cases [4].

Management and treatment of LVNC are centered on the prevention of major complications, namely, stroke and sudden cardiac death [10]. ICD is indicated for primary prevention in patients with LV ejection fraction of up to 35% and functional class II or HF (New York Heart Association), for secondary prevention, or in patients who have an additional risk factor, such as the family history of sudden death, nonsustained ventricular tachycardia, or a history of syncope [3]. Symptoms of HF should be treated according to a guideline-directed medical therapy in the setting of systolic and/or diastolic dysfunction [10].

Prognosis is heterogeneous, and HF, ventricular arrhythmias, and systemic embolisms are the most frequent cardiovascular complications [1]. LVEF < 50% and noncompaction extending from the apex to the mid or basal segments seem to be associated with higher all-cause mortality [7]. Although, the degree of hypertrabeculation has not been associated with either LV remodeling or outcome [1].

## Conclusions

LVNC is a genetic disease with a wide variety of clinical presentations. HF and cardiac arrest secondary to ventricular arrhythmias are severe manifestations of the disease and determine a poor prognosis. We highlight an interesting case of LVNC, which was treated with ICD for secondary prevention and combined medical therapy with ARNI, beta-blockers, and mineralocorticoid receptor antagonists, and was noted to have a good long-term outcome. However, per current evidence, it is still unclear which patients respond well to appropriate treatment, and it is difficult to predict improvement in cardiac function. We reported a brief discussion and literature review on the current evaluation, management, and prognosis of patients with LVNC.
